# Transactions of the Buffalo Obstetrical Society

**Published:** 1885-08

**Authors:** 


### TRANSACTIONS OF THE BUFFALO OBSTETRICAL SOCIETY.

*Stated Meeting, June* 23, 1885.

The President, Dr. W. W. Potter, in the Chair.

Dr. P. W. Van Peyma read a paper on "The Management of the Puerperal Breast."

He first reviewed the anatomy of the female breast as requisite to a proper understanding of its histology and physiology.

The human breasts rarely exceeded two in number, though in a few instances supernumary glands have been observed. Supplementary nipples were less uncommon. These latter were situated a short distance from the principal one, were smaller, and yielded milk during lactation. In the lower animals, the number of nipples bore relations to the number of young at a birth; in the human family, this relation had not been traced. The developed gland was peculiar to the female, though in exceptional instances it had developed and secreted milk in the male. Secretion had been observed in the newly-born infant, and in the non-pregnant female.

The human breast was a racemose gland, composed of lobes, fifteen or twenty in number, these being sub-divided into lobules. Each lobule was formed of great numbers of acini or vesicles, opening into minute ducts. The ultimate ducts averaged 1-300 of an inch in diameter, which united to form larger ducts, and these again united into fifteen or twenty canals, corresponding to the separate lobes. These tubuli lactiferi converged towards the areola, under which they became dilated into sinuses of 1-6 to ¼ of an inch in diameter, and these continued, their lumen being contracted into the nipple, opening by orifices still more contracted. Connective tissue bound all these component parts together, acting as a frame-work and support to the blood-vessels, lymphatics, and nerves, ramifying through the gland. Involuntary muscular fibre was found in the duct walls; and it was arranged so as to form tissue analogous to the erectile tissue of the other generative organs.

The veins at the base of the nipple formed an anastomotic circle called the circulus venosus, thence ramifying to the circumference, emptying into the axillary and internal mammary veins. The arteries were derived from the intercostal and thoracic branches of the axillary; while the lymphatics, in great part, ran to the axillary glands, a few passing through the intercostal spaces to the mediastinal gland. The nerves were derived from the internal and lateral cutaneous nerves of the thorax.

The tubercles at the areola enlarged during pregnancy, and secreted sebaceous material, which served to protect the surface. The histological appearance varied greatly between the conditions of secretive activity and quiescence. During, or just previous to lactation, the vesicles were composed of a net-work of basement membrane, holding within its meshes from fifteen to twenty large polyhedral, usually pentagonal or hexagonal, epithelium cells, each containing a large nucleus, and occasionally a nucleolus. The cells of the ducts were pavement near their openings, but became more glandular towards the interior.*

The embryological origin of the mammary gland was still an open question. It was commonly supposed to have had its origin from the ectoderm or epiblast, by a complex extension downwards, beginning in the fifth fœtal month, as a solid rounded process from the mucosum. Creighton came to the conclusion that the secreting structure was formed in a layer of mesoblast beneath the outer skin, and that the ducts were essentially secondary formations, owing their existence to a force from within. But it was still a controversy whether the gland developed from a single center, or simultaneously from numerous points.

The physiology of the gland was limited to the maintenance of its growth, and the secretion of milk; and these two functions travelled much in parallel lines. The formation of

* This portion of the paper was illustrated by drawings made by the author.

milk was now known to depend on the activity of the large epithelial cells. Physically, this consisted in a process of vacuolation, by which the protoplasm becomes liquified, the nucleus pressed to one side, and after the formation of one or more globules, the liquefaction or rupture of the cell-wall, and escape into the cavity of the acinus of the liquid cell contents. The result of this process, when at its height, was a collection within the vesicles and ducts of perfect milk. The formation of the perfect nucleated epithelium, whose activity and changes resulted in the production of milk, occurred only at the time of lactation, or a short period antecedent thereto. When the action of the cell was imperfect, as at the beginning or end of lactation, albumen was in excess of casein; but as long as the cell possessed its proper activity, the formation of casein became prominent. Casein, it had been suggested, might be formed by splitting up of albumen by some fermentative process, but such ferment had not yet been isolated. That milk-sugar was also formed by the protoplasm of the cell, was indicated by the fact that the sugar was not dependent on carbohydrate food, but was maintained in abundance in the milk of carnivora when they were fed exclusively on meat, as free as possible from any kind of sugar or glycogen. We thus had evidence of the formation, by the direct metabolic activity of the secreting cell, of the representatives of the three great classes of food stuffs—proteids, fats and carbohydrates—out of the comprehensive substance, protoplasm; and what we saw taking place in the mammary gland was probably a picture of what was going on in all protoplasmic bodies.

Were the fat of the milk not ejected from the mammary cell, the gland would become a mass of adipose tissue, especially if, by a slight change in the metabolism, the production of fat were exalted at the expense of the production of casein or milk-sugar. If, again, by a similar slight change, the milk-sugar were accumulated rather than the fat or proteid, we should have a result which, by an easy step, would lead us to glycogenic tissue. Finally, if the proteid accumulations were greater than the fatty or the saccharine, these being carried off in some way or other, we should have an image of the nutrition of an ordinary nitrogenous tissue.

The presence of nervous and muscular tissue in the breast, made the influences of the emotions over the quantity and character of the secretion of easy explanation.

The diseases of the breast of interest in this connection were numerous. The amount of secretion might be either scanty or profuse, and, strangely enough, the same general remedies were useful in both conditions. In proportion as the secretion became inordinate in quantity, the quality deteriorated; thus, this condition, as well as its opposite, was benefitted by generous diet and attention to the general health. In the former condition as well as in galactorrhœa pressure, by means of a sponge and bandage, the sponge being moistened with some astringent, as lead wash, etc., might be employed. Belladonna and camphor should be used with caution, lest the secretion be completely suspended. Among the diseases of the parturient breast, excoriations, chapps, and fissures of the nipples; obstructions to the flow of milk, and consequent engorgement; inflammation, and abscess were some of the most important.

The causes were varied. Among the more common were exposure to cold, injury, and to the irritation of substances coming in contact with the nipple. Previous disease of the gland, and a low state of health, were predisposing causes. The prophylactic measures employed during pregnancy were well known. These included exposure to air, bathing, stimulating or astringent washes, suction of the nipples when too small, etc. Ordinarily, the child should be applied to the breast as soon as the mother had sufficiently recovered from the exhaustion of labor. Regular intervals for nursing should be maintained when practicable, without too arbitrary determination, at about three hours or less, with a gradual lengthening of the period as the age of the child increased. As a rule, weaning should not be postponed beyond one year from the commencement of lactation, and should be insisted on upon the occurrence of menstruation or pregnancy. Cleanliness of the nipples and the preservation of the general health were important prophylactics. A more or less general engorgement was not to be confounded with inflammation, to which it, later, might give rise. This quite common condition only required increased suction for its subsidence.

For the various abraded and fissured conditions, the nipple shield afforded an important means of relieving the strain of nursing. The intervals between nursing might be advantageously prolonged, by nursing the breasts alternately at protracted periods.

Inflammation of deep portions of the gland was often caused by extension from external fissures. Support of the breast was often an important consideration in prophylaxis. He concluded the essay by quoting the words of Dr. Harris: "There are probably few physicians who have not felt the need of more certain methods for averting inflammation and suppuration of the puerperal breast. The use of very gentle friction with oil, the withdrawal of milk in sufficient quantities to relieve distension of the glands, belladonna plaster, cold applications, hot fomentations, poultices, the local treatment of sore or fissured nipples, supporting the breast in a sling, abstinence from fluids and liquid food, the internal administration of salive laxatives, iodide of potassium, quinine, phytolacca decandra, and belladonna, have long been considered measures more or less important in the treatment of this painfully wearing affection."* The view that the retained milk, undergoing some fermentative change, acted as an irritant, was one very generally accepted. Physicians, therefore, generally agreed in the importance of relieving the breast; they were also equally agreed that the retained secretion soon became unfit as food. If, as was now claimed, these views were erroneous, the way was cleared to a very important advance in therapeutics. The injury resulting

*American Journal of Obstetrics, January, 1885.

from continuous nursing was manifest. Dr. Harris, in the paper just quoted from, advocates a method of bandaging by which he supports the breast, and exerts gentle pressure at the same time. The nipple was left free, and the drainage of milk allowed to follow its own course. The patient, under this plan, required daily observation and an occasional re-application of the bandage. After a variable period, it was claimed that the inflammation subsided, when the child might be re-applied to the breast. Dr. H. supported these views by a report of cases. Of local blood-letting, the essayist had no knowledge. An abscess having formed, an incision at the most dependent point should be made. In the subsequent treatment of the cavity, besides general remedies, the bandage became especially useful in promoting more rapid contraction and healing.

DISCUSSION.

Dr. M. Hartwig spoke of the importance of studying the pathology of diseases of the breast, and said that the author's statement as to the nucleolus was new to him. Alluding to the curious anomolies in connection with this subject, he referred to the instance reported by Hyrtl, of a man's having nursed a child; and another where an infant suckled its grandmother. He thought the period of nursing should be lengthened, and believed it was proper to keep up breast-feeding until a child had sixteen teeth. He did not coincide with those who gave the child the breast early after delivery, but thought it better to wait three or four days. He believed it was conducive to sore nipples for the child to constantly draw at the breast before the milk appeared. Abscesses were produced by germs entering the cracks or fissures, and not from distension of milk ducts by hypersecretion or failure to empty them. Abscesses were generally opened too late, or later than they should have been; though it was well not to commit the opposite error by opening them too early. The skin does not need to be red to indicate pus; so, too, there need not always be pain.

Dr. R. L. Banta referred to the treatment of mastitis as described in the paper of Dr. Harris, which aimed to secure rest from pain, secretion and motion. He had recently treated a case after this plan, but failed to obtain as much benefit as he had been led to expect from the cases reported in the article referred to. He believed that cases where abscesses formed were generally of the so-called scrofulous diathesis, and required constitutional as well as surgical and local treatment.

Dr. U. C. Lynde, present by invitation, said he had seen a case of development of the mammary gland in the male, but had never had the opportunity of observing a supernumary nipple or breast. Commenting on the pathology of diseases of the breast, he referred to the laws governing secretion and excretion, and thought the latter term should be confined to the elimination of effete material from the economy, such as urea by the kidneys, etc. In regard to the treatment of mastitis, he had employed nearly all plans, and he might say he had failed with all remedies on occasions, excepting the fluid extract of poke root. This drug he gave in ten-drop doses every hour, and had come to regard it as a specific in the arrest of the suppurative process, and the control of pain. He did not think that mastitis always originated in the introduction of germs through the nipple-cracks. In regard to the practice of giving the child the breast early, he regarded it as beneficial to the child, and was not prepared to say that it was harmful to the mother. There might, however, be cases where it would work perniciously in both directions.

Dr. A. Dagenais, also present by invitation, in commenting upon the treatment of the puerperal breast, said he was prepared to endorse all Dr. Lynde had said in regard to the use of poke root. He had, however, been in the habit of giving it in somewhat larger doses. These affections he regarded as often of the most perplexing nature to deal with, and any hints or suggestions which could be given were of great value. He looked favorably upon the support and elastic-pressure method of treatment, and also favored the early evacuation of pus in cases where suppuration took place.

The President had been much interested in the histological review which the essayist had given, but was in hopes to have heard more from the practical experience of the writer on the subject of treatment. He regarded it as one of the severest strains upon good nature or amiability of temper, to care for one of these cases when the infant, as would sometimes happen, obstinately refused the breast. To make the child finally nurse in such a case, was likely to solve the whole problem of health to both mother and infant. Among other things in the treatment of cracked nipples, he had found it a good plan to treat the child's mouth; for it would frequently be found that the salivary secretion acted as a poison or irritant to the raw tissues. This would prove important even though there was no stomatitis or other *apparent* disease of the mouth. It was his custom to direct the application of the child to the breast as soon as possible after delivery, that it might cultivate the habit of suckling at once; for it was often difficult to establish it after a few days of spoon-feeding. He did not see how this plan was more likely to produce fissures, and he thought it beneficial to the child to ingest the colostrum as soon as obtainable.

